# Strategies for successful recombinant expression of disulfide bond-dependent proteins in *Escherichia coli*

**DOI:** 10.1186/1475-2859-8-26

**Published:** 2009-05-14

**Authors:** Ario de Marco

**Affiliations:** 1 Cogentech, IFOM-IEO Campus for Oncogenomic, via Adamello, 16 - 20139, Milano, Italy

## Abstract

Bacteria are simple and cost effective hosts for producing recombinant proteins. However, their physiological features may limit their use for obtaining in native form proteins of some specific structural classes, such as for instance polypeptides that undergo extensive post-translational modifications. To some extent, also the production of proteins that depending on disulfide bridges for their stability has been considered difficult in *E. coli*.

Both eukaryotic and prokaryotic organisms keep their cytoplasm reduced and, consequently, disulfide bond formation is impaired in this subcellular compartment. Disulfide bridges can stabilize protein structure and are often present in high abundance in secreted proteins. In eukaryotic cells such bonds are formed in the oxidizing environment of endoplasmic reticulum during the export process. Bacteria do not possess a similar specialized subcellular compartment, but they have both export systems and enzymatic activities aimed at the formation and at the quality control of disulfide bonds in the oxidizing periplasm.

This article reviews the available strategies for exploiting the physiological mechanisms of bactera to produce properly folded disulfide-bonded proteins.

## Background

**Figure 1:**
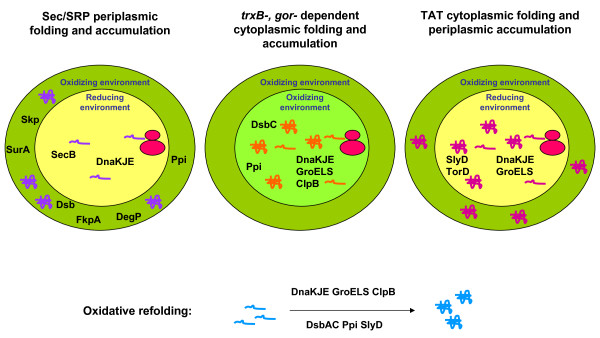
**Schematic representation of folding pathways and cellular localization of proteins that depend on oxidative environment to reach their native structure**. Unfolded proteins can be translocated into the periplasm post-translationally (Sec mechanism) or co-translationally (SPR mechanism), according to their specific leader sequences. SecB is the physiological chaperone involved in stabilizing unfolded proteins bound to Sec translocation machinery, but the activity of other cytoplasmic chaperones like DnaK can be beneficial. Once in the periplasm proteins folding is mediated by the Dsb oxidases/isomerases, by chaperones such as Skp, DegP and FkpA, and by peptidyl-prolyl isomerases such as SurA, PpiA, and PpiB. Double mutant strains, in which the thioredoxin/glutaredoxin reductase (*trxB*^-^, *gor*^-^) pathway is silenced, are characterized by the presence of an oxidizing cytoplasm, compatible with the folding of proteins that need disulfide bridges for stabilizing their structure. Cytoplasmic accumulation of correctly folded disulfide-dependent proteins is improved by disulfide isomerase DsbC co-expression and cytoplasmic chaperone (DnaKJE, GroELS, ClpB). Finally, proteins provided with a Tat export leader sequence first fold in the cytoplasm and then are tanslocated into the periplasm. Chaperones like DnaKJE, can stabilize the precursor in the cytoplasm and chaperones such as SlyD and TorD support the pathway efficiency probably binding to the export leader peptide.

The success of recombinant protein expression in *E. coli *depends mainly on the capability of avoiding unproductive interactions of newly expressed polypeptides. Such interactions lead to aggregation of folding intermediates instead of yielding native proteins. The efficiency of the process can be increased by favoring conditions that stabilize folding intermediates and promote the formation of mature structure. Several strategies may help in preventing protein aggregation by masking hydrophobic patches on their external surfaces. These include the introduction of chaperone molecules, adding detergents, or co-expressing interacting sub-units of larger complexes. Once the conditions have been optimized for keeping the folding intermediates monodispersed, it becomes crucial to speed up the folding process to reach stable native structures and avoid the accumulation of metastable configurations that remain potentially prone to aggregation. Foldases and isomerases may strongly enhance the folding (Fig 1).

The attention of this review will be focused on the technically available solutions to improve the bacterial expression of proteins that rely on disulfide bond formation to reach their native state. Such cys-cys bridges block folding units into stable conformations by linking residues in a covalent manner and their formation is necessary for a protein to achieve its stable tertiary structure.

The equilibrium between reduced and oxidized cysteines is regulated by the redox conditions of each cell compartment. In eukaryotic cells, the oxidative environment in which disulfide bonds are preferentially formed is the endoplasmic reticulum (ER). Therefore, polypeptides expressed in the reducing cytoplasm need to be directed to ER to complete their folding. The correct targeting to the subcellular compartment is mediated by signal peptides fused to the protein amino terminus that are removed after the import into the organelle. Prokaryotes share with eukaryotic cells the reducing cytoplasm, but do not have structures resembling the ER. Instead of it, they possess an oxidizing periplasm to which pro-peptides with an extra N-term export peptide can be translocated. Therefore, eukaryotic protein expression in bacteria periplasm is possible following the substitution of the ER with a bacterial signal sequence for periplasm translocation.

Alternative strategies consider promoting the formation of disulfide bonds by targeting the nascent polypeptides to the external medium or by modifying the redox state of cytoplasm to reach a mild oxidative environment (Figure 1). Both overexpression and direct fusion to chaperones, foldases, and stabilizing carriers has been tested for improving the yields of functional target proteins.

**Figure 2:**
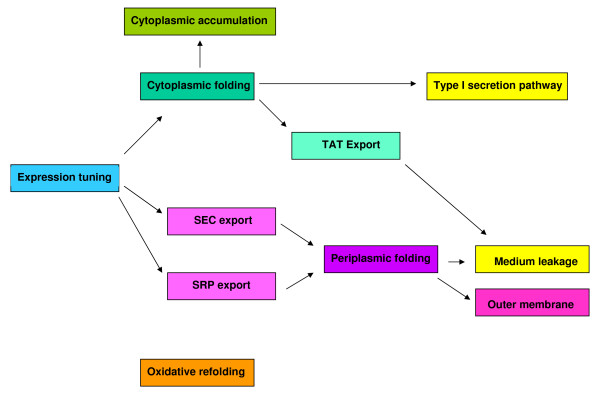
**Flow-chart summarizing the different possibilities for producing disulfide-dependent proteins in bacteria**. Expression is optimized and protein folding directed either in the cytoplasm or in the periplasm. Once folded in the cytoplasm, proteins can accumulate in the same cell compartment, or be exported to the periplasm by the Tat-pathway, or be secreted to the medium by the type I secretion system. Unfolded proteins can be translocated post-translationaly (Sec) or co-translationaly (SPR) into the periplasm and accumulate there, leak to the medium, or be exposed on the outer membrane. Precipitated proteins can be recovered into native structure by means of oxidative refolding.

Finally, protein aggregates can be first dissolved in chaotropic solutions to reach monodispersity and later be used as a starting material for oxidative refolding processes. A flowchart of the different alternatives is reported in Figure 2.

## Periplasmic expression

The most intuitive method to exploit *E. coli *for recovering folded disulfide-bond dependent recombinant proteins is to direct the translated polypeptides to the bacterial periplasm. There are clear physiological reasons for such an approach: the periplasm, in contrast to the cytoplasm, is an oxidizing compartment and it hosts enzymes catalyzing disulfide bond formation and their isomerization, as well as specific chaperones and foldases [1-3]. However, the necessity of translocating nascent polypeptides through the inner membrane introduces a delicate step since there is only a limited number of available gates for reaching the periplasm and metastable precursors may consequently accumulate in the cytoplasm.

### Modulation of the expression level

Considering the potential danger of having an overcrowded cytoplasm, some authors proposed that protein secretion could be effectively modulated at the translational level by modifying the Shine-Dalgarno sequence [4]. Other authors found that the crucial tuning region starts upstream of the Shine-Dalgarno region and spans approximately twenty nucleotides downstream of the initiation codon (translational initiation region) [5]. Coherently, also other parameters influencing the expression rate have been evaluated in relationship with the production of disulfide-bond dependent proteins, such as growth medium, plasmid origin of replication, and expression promoters. For instance, the expression features of tac, uspA, uspB, T7, trc, lacUV5, malK, pm/xylS have been investigated to achieve high product yields by preventing protein precipitation and cell lysis [6-12]. However, data have been collected under different experimental conditions and, therefore, they are not comparable, preventing the identification of surely preferable systems.

### Choice of the suitable secretion leader peptide

In the absence of an N-terminal signal peptide for periplasmic secretion, recombinant polypeptides expressed in bacteria accumulate in the cytoplasm. The fusion to suitable leader peptides allows for the translocation of unfolded precursors into the periplasm by either the Sec (relatively slow, post-translational translocation) or the SRP (fast, co-translational translocation) system [13,14], even in the case of aggregation prone proteins such as PNGase and large molecules like full-length immunoglobulins [15,16]. The search for optimal leader peptides to use in combination with recombinant proteins has been initially undertaken by comparing the efficiency of natural signal sequences identified in the precursors of bacterial periplasmic proteins, including the leader peptides from *spA, phoA, ribose binding protein, pelB, ompA, ompT, dsbA, torA, torT*, and *tolT*. Furthermore, both synthetic sequences and the phage pIII leader peptide were used [6,9,17-22]. Initially, the approach was not systematic and no clear preference for any among them was apparent, although *ompT *resulted preferable when coupled to overexpression of chaperones involved in the stabilization of intermediates translocated through the Sec export machinery [23].

However, a wide survey performed by Beckwith and co-workers identified a strong correlation between hydrophobicity of the leader peptide and export mechanism [24]. Apparently, cotranslational translocation by SRP needs the presence of highly hydrophobic leader sequences, even though further unknown biophysical features may be critical. The physiological necessity of the SRP pathway as an alternative to the post-translational secretion meditated by the Sec route is required to avoid premature folding of the proteins in the cytoplasm. The biotechnological implication of these conclusions is that poor periplasmic accumulation of rapidly folding recombinant proteins may be the consequence of their non-productive cytoplasmic (mis)folding that prevents efficient translocation and correct periplasmic folding. Therefore, the choice of the leader peptide may make the difference in terms of secretion efficiency, as demonstrated for thermodynamic stable proteins [25,26].

### Mechanism of protein oxidation in the periplasm

**Figure 3:**
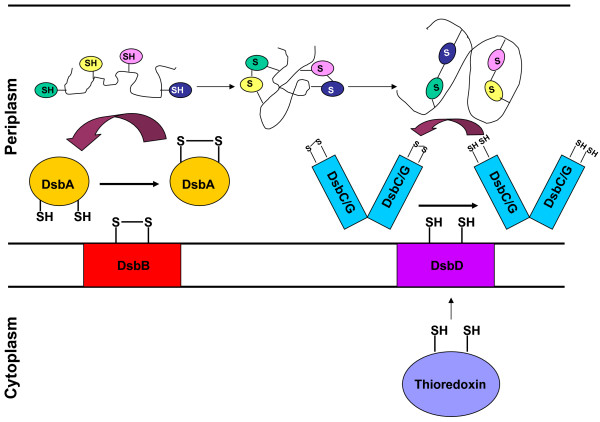
**Dsb-dependent protein oxidation and isomerization in the bacterial periplasm**. DsbA couples consecutive cysteines providing the disulfide bond and is re-charged by the inner membrane DsbB. Incorrect disulfides of oxidized proteins are scrambled by DsbC and DsbG. These isomerases are kept reduced by the inner membrane DsbD that, in return, is reduced by cytoplasmic thioredoxin.

Spontaneous protein oxidation in extracytoplasmic compartments is extremely slow and is incompatible with cell activity. Therefore, it is necessary that the disulfide bond formation is enzymatically catalyzed. Periplasmic protein oxidation is regulated by the five members of the Dsb protein system (DsbA, B, C, D, G) [1,2]. With the exception of DsbB, these proteins belong to the thioredoxin protein superfamily and are involved in both disulfide-bond formation and rearrangement. Specifically, the soluble monomer protein DsbA donates its disulfide bond to newly synthesized polypeptides (Figure 3) that are folding intermediates and need disulfide bonds to reach their native structure. Interestingly, DsbA binds its substrates by hydrophobic interactions using a chaperone-like recognition mechanism specific for partially unfolded proteins [27]. Such substrate-independent chaperone-like activity seems to have a general stabilizing effect on passenger proteins fused to DsbA mutant deprived of its oxidoreductase activity and, therefore, it has been successfully used as fusion partner even for the production of cytoplasmic proteins [28].

DsbA active site is regenerated by the transfer of electrons to the DsbB integral membrane protein, whose activity is crucial in maintaining DsbA in its active state (Figure 3) [27,29-32]. The DsbA/DsbB mediated oxidative process is very efficient, but may result in incorrect cysteine pairing and in trapping the target protein in non-native conformations. Therefore, a quality control mechanism for the rearrangement of the disulfide bonds was initially postulated and later identified. DsbC and DsbG are the isomerases that scramble incorrect cystin bridges and, therefore, play a key-role in the periplasmic protein folding process (Figure 3) [33-36]. The active cysteines of DsbC and DsbG must remain reduced in an oxidizing environment to be functional. This condition is achieved by the action of the integral inner membrane DsbD. Such protein constantly reduces the isomerases by transferring to them the electrons made available by the cytoplasmic thioredoxin [37-41]. Similarly to DsbA, both DsbC and DsbG have chaperone activity. Since misfolding could be a direct consequence of incorrect disulfides, DsbC/DsbG chaperone activity favors the recognition and interaction with substrates necessiting disulfide isomerization [1,42]. Such homodimer isomerases are V-shaped and use the structure flexibility of the cleft embodied by the two arms to bind unfolded structures of various sizes. Their adaptability allows the recognition of largely heterogeneous substrates and explains the elevated efficiency of bacterial DsbC/DsbG in isomerasing also heterologous proteins [1].

Although the two routes of keeping DsbA oxidized and DsbC/DsbG reduced have been always considered strictly separated [1], recent data indicate a possible direct cooperation between DsbA and DsbC [43], and chimeras of DsbA and DsbC proved to be able to serve both as oxidase and as isomerase, thus reconciling the competing pathways leading to DsbA oxidation and DsbC reduction [44]. Furthermore, it has been demonstrated that DsbA can be mutated to become an isomerase by adding a linker with peptide binding capacity and that provides a dimerisation domain. These characteristics prevent its immediate oxidation by DsbB and allow substrate recognition and binding [45]. Such results will probably simplify the biotechnological applications of Dsb proteins *in vitro*.

### Use of Dsb proteins for biotechnological purposes

The relevance of the Dsb protein family for the correct folding of disulfide-bond dependent proteins in the *E. coli *suggested that boosting such molecular machinery could result in increased yields of correctly folded recombinant constructs. The accumulation of a functional scFv was positively affected by co-expression of DsbA, B, C, D [46], both DsbAB and DsbCD overexpression improved the production of active horseradish peroxidase [47], whilst co-expression of DsbA and DsbC increased the yield of functional human plasma retinol-binding protein [48]. Fusion to DsbA increased the accumulation of few mg/L culture medium of active bovine enterokinase and human pro-insulin, a polypeptide stabilized by three disulfide bonds [49,50]. However, in the case of Ragi bifunctional inhibitor, a protein with five overlapping disulfide bonds, DsbA activity resulted in the accumulation of non-native intermediates, whilst the availability of DsbC significantly increased the yield of the native protein both *in vitro *and *in vivo *[51]. Again, the overexpressoion of DsbA, B, C, D and DsbCD, but not DsbAB, increased the accumulation of active nerve growth factor beta [52] and DsbC was the key foldase also for enhancing the production of horseradish peroxidase and brain-derived neurotrophic factor, although its synergistic effect with DsbA, B, and D was detectable [53,54]. These results were confirmed by experiments performed with plasminogen activator and a scFv [55,56] and seem to indicate that the isomerase DsbC activity could be the limiting factor for the correct folding of at least part of the recombinant proteins expressed in the *E. coli *periplasm.

The different responses observed when using eukaryotic substrates and DsbA or DsbC overexpression might be explained by examining the structure of each single substrate protein. DsbA couples cysteines as soon as they are available and, therefore, only disulfides between consecutive residues are formed. As a consequence, DsbC activity is not necessary for proteins in which only disulfide bonds between consecutive cysteines are present in the native structure [57]. In contrast, bond scrambling is DsbC-dependent. The correlation between the presence of nonconsecutive disulfides and DsbC-dependence for achieving the correct folding has been demonstrated for eukaryotic as well as for bacterial proteins [51,58]. However, although the bacterial RNaseI is a natural DsbC substrate with three consecutive and one nonconsecutive disulfide bonds [59], it is possible to artificially tune the periplasm redox potential to achieve conditions that allow DsbA to correctly catalyze the bond formation between the nonconsecutive residues [60].

The pivotal role of thiol-disulfide oxidoreductases in supporting the correct folding of disulfide-bond dependent proteins is conserved in Gram-negative bacterial periplasm [61]. Interestingly, functionality seems to be retained also by phylogenetically distant proteins, such as endoplasmic reticulum disulfide isomerase from mammalian and yeast since, despite the low sequence homology, a marked structural similarity exists [62-64]. This observation led to successful engineering of human disulfide isomerase in the *E. coli *periplasm and even in the Gram-positive *Bacillus brevis*, with consequent functional restoration in *dsbA *mutants and yield improvement of functional proteins [65,66].

### Coexpression of periplasmic chaperones and foldases

The process leading to correct protein folding in the *E. coli *periplasm is mediated by the activity of proteins belonging to several classes and that often have partially overlapping functions (Fig 1). There are peptidyl-prolyl isomerases and chaperones such as SurA, FkpA, PpiA, PpiD, and Skp, and chaperone/proteases such as DegP [67-73]. FkpA and SurA overexpression contributed to an increase in the accumulation of functional human plasma retinol-binding protein [48], FkpA, DegP, DsbA, and DsbC rescued the activity of lipase B [74], and both the chaperone and protease activities of DegP were necessary to enhance the yields of penicillin acylase [71,75].

The prolyl-cis/trans isomerase activity of Ppi apparently catalyzes the rate-limiting step of the recombinant antibody folding represented by the isomerization of proline 95 (Kabat numbering) [76]. Its coexpression increased the solubility of scFvs and Fab fragments [77-79], but this enzyme was also successfully used as a fusion partner for recombinant antibody expression [4,80]. Similarly, fusions to FkpA resulted in increased yields of functional recombinant antibodies [81,82].

### Effect of cytoplasm chaperones on periplasmic protein accumulation

The mechanisms by which cytoplasmic chaperones can help periplasmic folding are less clear. In a few cases such molecules were directly secreted to the periplasmic space, as was the case of DnaJ and Hsp25 that increased the yields of native plasminogen activator [83]. Probably such molecular chaperones had an unspecific stabilizing effect on folding intermediates, but no sound rationale supports the strategy of boosting the periplasm with cytoplasmic chaperones, instead of using periplasmic ones. In contrast, the idea of overexpressing cytoplasmic chaperones in the cytoplasm might be justified by the consideration that nascent polypeptides are translocated as unfolded intermediates into the periplasm by the Sec/SRP systems and, therefore, their potential aggregation must be prevented (Figure 1). SecB is the natural chaperone that binds polypeptides to be secreted post-translationaly. Apparently, SecB possesses specificity for 9-residue sequence motifs enriched in aromatic and basic residues [84]. The presence of these motifs within recombinant proteins may considerably influence their chances to be correctly delivered to the periplasm by the SEC system. However, the overexpression of other chaperones could compensate for poor SecB binding to non-specific substrates. They might unspecifically recognize and stabilize unfolded polypeptides in the cytoplasm, as suggested by the observation that chaperone overexpression in the cytoplasm was beneficial for the accumulation of some periplasmic proteins [85]. Different combinations have been tested, such as DnaKJE (DnaK, DnaJ, GrpE) that increased the accumulation of functional scFvs against domoic acid and the mycotoxin deoxynivalenol [56,86]. DnaKJ co-expression prevented the formation of inclusion bodies of CorA and improved the periplasmic accumulation of granulocyte-colony stimulating factor [87,88]. In contrast, cytoplasmic chaperone overexpression failed to improve the yields of horseradish peroxidase [47].

The accumulation rate of SecB, the physiological chaperone for the Sec system, is apparently efficiently tuned by the effective amount of potential substrates and, consequently, its exogenous overexpression was not considered necessary [13,89,90]. However, in at least one case higher periplasmic accumulation of heterologous proteins was detected as a consequence of cytoplasmic SecB overexpression [91]. Other authors found that the overexpression of SecB and DnaKJ was beneficial in promoting recombinant accumulation of human proteins in the periplasm, but only in combination with signal sequence optimization [92].

### Advantages and limitations of fusion constructs

The problem of stabilizing the folding intermediates has been addressed also by fusing the target polypeptide to protein carriers. Periplasmic EETI-II, erythropoietin, and scFv production was increased by fusions with maltose-binding protein and immunoglobulin-constant domain, whilst human proinsulin and pepsinogen yields were enhanced by fusions with ecotin, a trypsin inhibitor that could favor target protein accumulation by reducing its degradation [93-96]. A double ecotin-ubiquitin tag has been recently engineered. It should allow both the stabilization of the passenger proteins and their recovery with their authentic amino terminus [12].

It must be underlined that protein fusion always carries an inherent risk of resulting in soluble but incorrectly folded target protein. For instance, fusions of maltose binding protein and some scFvs resulted in higher periplasmic yields, but the antibodies were not functional, whilst the fusion to alkaline phosphatase stabilized both production and activity [97]. Alkaline phosphatase has been often used as a fusion partner for recombinant antibodies. It is not only useful for enhancing the production but, dimerizing, it increases the avidity of the immunoproteins for their substrate and allows their direct detection by enzymatic assay [97-100].

Protein A or single and tandem repeats of its B- and Z-domains were successfully used for expressing and purifying a scFv and human proteins such as proinsulin and coagulation factor VII [9,101-105]. Fusion to barnase resulted in correct folding of ICK and McoEeTI, but the construct was directed to the culture medium [106,107].

Other fusion partners, especially in combination with recombinant antibodies, were considered not for facilitating folding and increasing yields, but to obtain reagents more suitable for the final applications. A biotinylation-consensus sequence has been fused to Fab fragments to obtain streptavidin-affine immunoconstructs [108], GFP has been fused to scFvs for recovering immunuofluorescent reagents [109], and fusion to nuclear import/export sequences have been used to direct scFvs to specific subcellular localizations [110].

### Effect of chemical chaperones

A positive effect of low molecular weight additives (chemical chaperones) supplemented in the culture medium was sometimes observed in terms of yields of periplasmic expressed proteins. Sorbitol addition to the culture medium resulted in higher accumulation of a functional scFv [46], glycine betaine and sucrose were beneficial for the folding of immunotoxin and cytochrome c550 [111,112], whilst L-arginine and ethanol increased the yields of human pro-insulin, plasminogen activator, and a scFv [50,83]. Also the supply of reduced glutathione, alone or in combination with DsbC overexpression, increased the accumulation of disulfide-dependent proteins [51,83].

### Twin-arginine translocase pathway

Although the Sec and SRP systems are responsible for translocating most of the proteins into the periplasm, bacteria possess another mechanism -the twin-arginine translocase (Tat) pathway- that enables the transport of folded proteins across the inner plasma membrane (Figure 1) [113]. *E. coli *genome encodes some tens of proteins with a signal-peptide capable of interacting with Tat machinery [114] and such export route has been exploited for recombinant expression as well [115,116]. The overexpression of all the *tatABC *genes expressing the proteins involved in the transport increased the translocation of a fluorescent reporter [117]. Also the overexpression of the cytoplasmic chaperone DnaK seems particularly useful in increasing the efficiency of the Tat pathway at both physiological and protein overexpression-induced stress conditions [118,119]. Such effect could be a consequence of the DnaK role in facilitating the substrate folding into native structures. Another explanation considers that virtually all Tat leader peptides contain recognition sequences for DnaK [120,121]. Therefore, Santini et al. [122] proposed that DnaK sequesters the protein intermediates in the cytoplasm by masking their leader peptide until the folding is complete.

Other chaperone-like proteins as SlyD and TorD have been reported improving Tat efficiency [118,123,124]. Interestingly, scFvs correctly folded in the cytoplasm (intrabody mutants or wild type constructs expressed in *trxB*^-^*gor*^- ^cells) were efficiently transported by the Tat system [125,126].

### High throughput selection of suitable clones

Protein accumulation varies from cell to cell inside a population and, therefore, strategies for selecting the most productive clones are relevant. Similarly, it may be necessary to identify, within large bacteria cultures expressing clone libraries, the few cells producing proteins/antibodies with desired features. Flow cytometry represents a powerful methodology since it enables a quick and thorough screen of large numbers of constructs and it has been successfully applied for selecting proteins, recombinant and full-length antibodies accumulated in the periplasm [127-132]. Several approaches have been described, all referring to the same principle of labeling proteins accumulated in the periplasm to enable the isolation of the productive bacteria. In the simplest protocol, proteins accumulate directly in the the periplasm, otherwise recombinant antibodies are anchored to the periplasmic side of the inner membrane through a lipoprotein domain, or full-length antibodies, secreted and folded in the periplasm, are captured through their Fc domain by an anchoring fusion construct consisting of the Z-domain of the Protein A and an inner membrane lipoprotein [129,132]. The outer membrane is successively permeabilized to form spheroplasts and the recombinant proteins/antibodies are labeled by binding to fluorescent antibodies/antigens. Finally, the cultured bacteria are sorted by flow cytometry according to the expression and affinity of the produced constructs. Such approach may become a paradigm for efficiently screening tagged proteins and antibodies expressed in the periplasmic space.

### Periplasmic inclusion body formation

Despite the periplasmic quality control and the observation that the FkpA chaperone activity seems to be effective in suppressing inclusion body formation [133], proteins can aggregate also in this cellular compartment. The simple overexpression is already sufficient to induce the accumulation of the bacterial beta-lactamase protein inclusion bodies into the periplasm [134,135]. Similarly to what was observed in cytoplasmic aggregates, the proteins trapped in periplasmic inclusion bodies may partially preserve their physiological activity [135] and induce stress response [136]. There are relatively few papers reporting the accumulation of heterologous protein inclusion bodies in bacterial periplasm [8,75,137-139]. However, the number might be underestimated because cell fractionation is rarely undertaken and negative results are often not communicated. From the available information it seems that construct instability is often present in engineered polypeptides. For instance, both the addition of unpaired cysteines and sub-optimal length or composition of the linkers connecting the variable domains may be strongly detrimental for the scFv yields [140,141]. Jeong and Lee systematically tried to alleviate heterologous protein precipitation in the periplasm by analyzing the effect of bacterial strain, growth temperature, expression vector features (signal peptide, promoter, codon usage optimization, linker compositions), and DsbA co-expression, but general conclusions require further studies [8,142].

### Induced accumulation of periplasmic proteins into the culture medium

The recovery of periplasmic proteins is usually achieved by disruption of the outer membrane by osmotic shock. Such purification protocol has the advantage of limiting the contamination with cytoplasmic material. However, as an alternative it has been also proposed to induce the lysis of the outer membrane to recover the target protein directly from the culture medium avoiding a cumbersome purification step (Figure 2). The most investigated solution is based on the expression of Kil protein, a bacteriocin that induces periplasmic protein release through induced membrane solubilization [143,144]. Lysis can be induced by regulating the Kil protein expression with a stationary-phase or a temperature inducible promoter [145,146] and the strategy was successfully used to recover from the medium interleukin-2, beta-glucanase, and streptavidin [144,145,147]. Also the co-expression of the TolA protein has been proposed for inducing the external membrane permeabilization [17].

The accumulation of proteins in the culture medium, and specifically of recombinant antibodies, expressed for being secreted into the periplasm has been often observed [148,149]. The reasons remain unknown and cell lysis does not seem to be always involved in the protein leakage. However, it has been shown that several different factors such as growth conditions, inducer concentration, and aminoacid substitutions can tune the leakage of scFvs and have been proposed as means to regulate scFv partition between periplasm and medium [149,150]. The influence of the expression level on protein leakage from the periplasm to the growth medium has been directly demonstrated comparing different promoters [10].

## Cytoplasmic expression

Early observations indicated that the disulfide bridges in both β-lactamase and alkaline phosphatase precursors expressed in *E. coli *were formed only after their translocation and processing in the periplasm and that it was possible to prevent their oxidative folding by trapping the precursors in the cytoplasm [151,152]. These results were explained by the presence of a reducing cytoplasm and an oxidizing periplasm in bacteria. Furthermore, they indicated that no protein requiring the formation of disulfide bonds for reaching its native structure can be produced in a functional form in the cytoplasm. The correlation between presence of disulfide bonds and native, functional structures was exploited by Beckwith and co-workers in their pioneering attempt to identify mutations that enabled the formation of active alkaline phosphatase in bacteria cytoplasm. It turned out that alkaline phosphatase folded correctly when expressed in bacteria hosting mutations that blocked the reduction of cysteines in the cytoplasm by silencing the activity of the thioredoxin reductase [153]. The preliminary model indicated that NADPH was the source of reducing potential used by thioredoxin reductase to reduce oxidized thioredoxin. However, the data suggested that another thioredoxin-like protein could be involved in a parallel reducing route. The search for complementary reducing mechanism(s) led to the identification of a second thioredoxin and of glutathione oxidoreductases, all involved in the mechanism aimed at reducing cytoplasmic cysteines [154,155]. The apparent redundancy of the reducing machinery is probably due to the necessity of obtaining the maximal electron transfer efficiency between protein pairs with varying redox potentials [156]. Under physiological conditions the directionality of the electron flux is maintained. However, it was shown that thioredoxin acts as a reductase only when it remains constantly reduced by the thioredoxin reductase activity. In contrast, it can catalyze substrate oxidation when exported to periplasm and oxidized by DsbB [157,158]. Also glutaredoxin 3 can catalyze disulfide bond formation in the periplasm, but its activity depends on oxidized glutathione availability rather than DsbB [159]. A similar process has been already demonstrated in eukaryotic cells [160] and its existence may also be hypothesed in the bacterial cytoplasm wherethe environment becomes oxidized as a consequence of impaired reducing activities.

Summarizing, it is possible to obtain an oxidizing cytoplasm in a cellular system in which glutaredoxin activity is abrogated by *gor*^- ^mutation, and both thioredoxin 1 and 2 are kept oxidized as a consequence of thioredoxin reductase mutations (Figure 1). Reduced glutathione, necessary to preserve cell viability, is produced by the disulfide reductase activity of mutated peroxiredoxin AhpC [161,162]. Interestingly, AhpC reductase activity does not significantly influence the oxidizing condition of the cytoplasm in *trxB*^-^, *gor*^- ^cells [162]. Such observation prompted to investigate the biotechnological potential of both single (*trxB*^-^) and double (*trxB*^-^, *gor*^-^) mutant strains -now commercialized with the names of AD494 and Origami (Novagen), respectively- for the cytoplasmic expression of recombinant proteins with multiple disulfide bonds in their native structure. The encouraging data obtained in the first attempts [163] were further improved by combining cytoplasmic oxidation conditions and cytoplasmic accumulation of DsbC isomerase (Figure 1) [164].

Recently, a new expression strain has become available. SHuffle (New England BioLab) is *trxB*^-^, *gor*^- ^as the commercial Origami (Novagen), but overexpresses cytoplasmic DsbC and is spectinomycin selectable, allowing the transformation with the majority of the currently used vectors. Although no literature is available so far, it is expected that the SHuffle combination of oxidizing conditions and isomerization capability would strongly improve the correct folding of disulfide bond-dependent proteins in the *E. coli *cytoplasm.

The method of expressing disulfide-bond-dependent proteins in oxidized cytoplasm was validated by several groups and progressively optimized. The collagen prolyl 4-hydrolases yield was higher in the cytoplasm of *trxB*^-^, *gor*^- ^bacteria than in the periplasm of the corresponding BL21 wild type strain [165]. The oxidized cytoplasm allowed the accumulation of functional IgG-like extracellular domain receptor [166], Ig2 domain of neurolin [167], lipase B [168,169], chitinase [170], and anti-freeze proteins [171]. Furthermore, the correct formation of disulfide bonds in the cytoplasmic milieu has been proved in recombinant oxalate oxidase [172], peanut allergen Ara h 2 [173], and *Stereum purpureum *endopolygalacturonase [174].

### A specific class of disulfide-bonded proteins: the recombinant antibodies

As a consequence of the constantly increasing importance of recombinant antibody expression, a significant effort has been dedicated for optimizing their production in bacteria. Although few antibodies are stable in the absence of disulfide bonds and can be directly expressed as intrabodies in the reducing cytoplasm of host cells [175-179], the structure and the functionality of most of them strictly depends on correct cys-cys bridges.

Recombinant antibodies in scFv format have one intra-chain disulfide bond for each variable region, those in Fab format have a further inter chain bond. A scFv fragment fused to a consensus peptide for obtaining *in vivo *site-specific biotinylation, a bivalent Fv antibody fragment fused to molybdopterin synthase to induce its oligomerization, and some Fab fragments were successfully produced in the cytoplasm of *trxB*^-^, *gor*^- ^cells [180-182]. *Camelidae *recombinant antibodies in VHH format possess one intra-chain disulfide bond between framework residues and may have a further one to constrain the CDR3 loop. Also these antibodies and have been successfully produced in the cytoplasm of *trxB*^-^, *gor*^- ^bacteria [183].

### Stabilizing role of chaperones and DsbC isomerase

Chaperones and isomerase can facilitate the target protein folding by impairing non-productive aggregation of folding intermediates and have been often co-expressed. The cytoplasmic overexpression of the periplasmic chaperone Skp and of DsbC isomerase, as well as of the molecular chaperones GroELS and trigger factor, significantly improved the yields of recombinant antibodies [184], whilst the cytoplasmic co-expression of Skp and FkpA were ineffective in rescuing fibrolase venom, in contrast to the beneficial effect of DsbC [185]. The work of other groups confirmed that the cytoplasmic co-expression of DsbC in *trxB*^-^, *gor*^-^bacteria was advantageous for the functional cytoplasmic accumulation of recombinant antibodies in both scFv and VHH format [183,186]. In such conditions the oxidizing environment favors the formation of the cys-cys bridges inside the polypeptide sequence and the isomerase apparently improves the yields of correctly folded antibodies by exchanging the cysteine residues involved in non-native pairing. The strategy of coupling cytoplasm oxidative conditions and isomerase activity was optimized by Yuan et al. [187] who overproduced thioredoxin fusions of calobin venom in the presence of cytoplasmic trapped DsbC. Co-expression of DsbC, GroELS and, at lower extent, of trigger factor resulted in higher yields of a functional scFv against the receptor c-Met [188], whilst the recombinant co-expression of a peptidyl-prolyl isomerase from *Pyrococcus *improved yields and functionality of recombinant Fab fragment [189]. A well-documented review addressing the specific problem of the recombinant antibody expression in prokaryotes has been published by Arbabi-Ghahroudi et al. [190].

### Thioredoxin overexpression

The cytoplasmic accumulation of thioredoxin as a consequence of its recombinant overexpression in wild type bacteria was proposed for increasing the yields of co-expressed eukaryotic proteins without disulfide bonds in their native structure [191]. Similarly, the precipitation of eukaryotic disulfide-bond independent proteins in the bacteria cytoplasm was prevented after their fusion to thioredoxin [192]. Cytoplasmic expression of thioredoxin fusions rescued also polypeptides that failed to correctly fold in the periplasm [187], plant viscotoxins, and porcine pepsinogen A, although these proteins contain multiple disulfide bridges [193,194]. The reasons as to why thioredoxin can stabilize its passenger proteins in wild type bacteria is not well understood, although its contribution to scFv folding might be due to its chaperone properties [195], since a catalytic cysteine thioredoxin mutant was still effective in supporting both disulfide bond formation and functionality of the target protein [196].

In contrast, there is a logical reason for overexpressing cytoplasmic thioredoxin in double mutant *trxB*^-^, *gor*^-^bacteria since such enzyme can act as an oxidant when it operates in an oxidized milieu [157]. Therefore, it can actively contribute to maintain oxidizing conditions in the cytoplasm. Fusions between recombinant scFvs and thioredoxin 1 expressed in *trxB*^-^, *gor*^- ^bacteria resulted in increased cytoplasmic yields [196], correct folding of a scFv against the c-Met receptor [188], and of the first domain of the multiple Kazal-type inhibitor LEKTI, a polypeptide that contains two disulfide bridges in its native structure [197]. Both the His- and GST-fusions of BSPH1, a protein containing 2 fibronectin type-II domains each consisting of 2 disulfide bonds, aggregated when expressed in the cytoplasm of *trxB*^-^, *gor*^- ^bacteria, but their fusions with thioredoxin resulted insoluble and active proteins [198].

### Carriers used to improve protein correct folding

Other proteins have been used as fusion partners and showed positive effects on folding and functionality of recombinant proteines accumulated in bacteria cytoplasm. Carboxyterminus fusions to maltose binding protein stabilized several scFvs independently on the cytoplasm redox conditions [199], although less efficiently than thioredoxin [196], whilst fusion to NusA was strictly necessary to yield another functional scFv and APRIL in the cytoplasm of *trxB*^-^, *gor*^- ^cells [200,201]. Sumo has also been proposed as solubilizing carrier for disulfide-bonded proteins, but the structural quality of the recovered proteins has not been investigated [202,203]. However, in a recent report it has been shown that a scFv against VEGF-165 was soluble and active when expressed fused to sumo [204].

### Minimal redox conditions compatible with disulfide bond formation

Although the rational background for the expression of disulfide-bond dependent proteins in double mutant strain *trxB*^-^, *gor*^- ^is generally accepted and such strain has been successfully exploited, alone or in combination with the expression of foldases/chaperones, the usefulness of the single *trxB*^- ^mutation remains unclear. The glutathione oxidoreductase is still functional in such bacteria and its activity should be sufficient to keep the cytoplasm partially reduced [156]. Consequently, disulfide bond formation should be at least partially impaired. In practice, only minimal amounts of all expressed scFvs remained soluble and functional in the cytoplasm of *trxB*^- ^(AD494) cells [109] and the advantage over controls was limited also in the presence of thioredoxin co-expression [205]. A direct comparison between AD494 and *trxB*^-^, *gor*^- ^bacteria clearly indicated an advantage in using the double mutant [196]. In contrast, the same cells resulted significantly more efficient than conventional XL-Blue cells in producing an anti-progesterone scFv [206]. However, data concerning the oxidation state of this antibody are not reported. Information concerning folding features or functionality is limited also in other cases in which proteins were successfully produced in the cytoplasm of AD494 cells. However, at least in the case of the scorpion neurotoxin Lqq-V and fibronectin II-2 domain from MMP-2, the proteins were both functional and correctly folded [207,208]. Furthermore, the *trxB*^- ^strain, in combination with DnaKJ co-expression, allowed the production of biological active human SPARC in the cytoplasm [209]. In the case of a serpin domain, AD494 cells did not improve the total amount of soluble recombinant protein accumulated in the cytoplasm with respect to wild type bacteria, but it was correctly folded and active, whilst the protein expressed in the control cells did not form the essential disulfide bonds [210]. GroELS co-expression further increased the serpin domain solubility. In some cases, the combination of AD494 cells and thioredoxin fusions significantly improved the expression of functional target proteins. *Leishmania *chitinase, the extracellular domain of human thyrotropin receptor, and the disintegrin domain of jararhagin venom were purified, in large amounts, as soluble and active proteins [211-213].

In contrast, it has been reported that a pro-urokinase fusion with thioredoxin expressed in AD494 (DE3) strain accumulated in inclusion bodies, even when the system was supplemented with disulfide isomerase and chaperonines [214]. However, there is scarce access to negative results that would help in understanding the factors regulating disulfide bond-dependent protein expression in the cytoplasm of wild type and mutant strains.

### Protein engineering

A time-consuming but definitive approach for avoiding trial-and-error attempts in identifying the optimal cytoplasmic expression conditions to yield functional proteins with disulfide bonds is the generation of mutants that maintain a stable structure even in the absence of cys-cys bridges [215]. Such protein engineering efforts were particularly successful in the case of recombinant antibodies. Various strategies of mutation and molecular evolution have been used to generate disulfide-independent stable scFvs starting from a reduction-sensitive precursor [175,216-218]. Intrabodies were recovered from libraries in which CDR variability was introduced by hypermutation of a naturally disulfide bond-independent VHH or scFv backbone [219,220], and by selecting natural intrabodies by biopanning [186,220-222]. Such molecules are valuable tools with great application potentiality for intracellular immunization [179,223,224].

The substitution of critical residues in both the framework and in the CDRs has also been used to improve the periplasmic accumulation of recombinant antibodies [150,225-227].

### Outer membrane-bound proteins

Expressing secreted proteins anchored on the external side of bacteria membranes [228] has been initially considered to render them accessible for external binders rather than to produce material for purification purpose. Avirulent bacteria displaying peptides corresponding to pathology-related antigen determinants on their surface were designed as vaccine tools [229-231]. They succeeded in stimulating immunological reactions [229-234], but the externally anchored peptides were not thoroughly characterized at structural level and, therefore, it cannot be ruled out that immunoresponse was promoted by partially unfolded antigen domains.

However, more recent data show that correctly folded fusions of outer membrane transporters and both scFv and VHH recombinant antibodies were efficiently translocated on the outer bacteria surface [235,236]. Once displayed, anchored proteins can be automatically released into the medium by cleavage of a specific sequence recognized by a membrane protease like OmpT [233,237].

Several bacteria transporter proteins have been used to develop vectors in which they are fused to the proteins of interest [238,239], such as FliC [240], pullulanase [241], OprF [242], OprI [243], PhoE [229], MisL [237,244,245], and cytolysin [234].

Exposing the protein to the outer bacteria surface is a particularly valuable approach when the proteins can be directly used in such format. For instance, enteric coronaviruses were successfully neutralized in cultured epithelial cells treated with *E. coli *expressing fusions of IgA protease beta domain from *Neisseria gonnorrhoeae *with specific scFvs [246]. An adhesion domain of intimin has been used as an anchoring partner for exposing both proteins and peptide libraries on the surface of the bacteria outer membrane. The resulting bacteria were suitable for cell sorting and used to identify epitope-specific antibodies [247,248].

### Type I (extra cellular) secretion pathway

The type I secretion pathway is used by Gram-negative bacteria to transfer toxins and exoenzymes provided of a carboxyterminus export signal directly from the cytoplasm to the external medium [249-251]. These proteins are devoid of disulfide bridges but the work performed by Fernandez and co-workers demonstrated that fusions of the secreted protein alfa-haemolysin C-domain and antibody fragments devoid of periplasm export signal peptide were efficiently secreted in oxidized and functional form [252-254]. Functional alfa-haemolysin fusions of both scFvs and VHHs were recovered from the culture medium [252-255] and hemolysin-fusion of Shiga toxin B subunit [245] was able to induce host immunoreaction in inoculated rabbits.

### Oxidative refolding of denatured proteins

Despite the great effort made by several groups for obtaining native proteins with correctly folded disulfide bridges in bacteria, many attempts failed and target proteins accumulated as precipitates. This observation prompted other groups to turn the disappointing results into an opportunity of pulling together large amounts of proteins and developing oxidative refolding strategies.

The methodologies available for protein refolding have been thoroughly reviewed [256-258] and here only parameters that are of specific interest for the disulfide bond-dependent proteins will be briefly presented.

Two common requisites for a refolding buffer are the presence of a redox couple and of alkaline pH. The redox couple (GSH/GSSG, MESNA/diMESNA, heteroaromatic thiols) is necessary to activate the cys residues [259-261], whilst the alkaline pH facilitates the nucleophilic attack-dependent disulfide bond formation [262]. Low temperature generally prevents non-productive contacts of metastable folding intermediates and can improve the final yields [263]. Non-productive interactions have been also successfully limited by modifying the reduced thiol groups before starting the refolding process. The cysteines of denatured proteins are first S-sulfonated or transformed into mixed disulfides and then the disulfide bonds are formed in the presence of a suitable redox system [259,264]. Surfactants and polyethylene glycol also showed stabilizing properties [265,266], whilst a dynamic redox environment, in which the conditions pass progressively from reductive to oxidative, has been proposed to maximize disulfide bond shuffling [267]. However, also simple dilution was effective for refolding heavy chain single domains [268].

An interesting recent paper shows the contradictory effect that stabilizing molecules may have on the refolding of disulfide-bond dependent proteins [269]. L-arginine is known to slow down the refolding process while suppressing hydrophobic interactions. Both these factors have been usually interpreted as an advantage for efficient refolding. However, although hydrophobic aggregation is prevented, the accumulation of intermediates with free cysteines leads to the formation of intermolecular disulfide bonds and the formation of progressively larger oligomers in an arginine-concentration dependent manner.

Productive oxidative refolding can be improved by different means. Chromatography allows separating the target protein from contaminants that can interfere during folding and has been successfully used to achieve the refolding of thioredoxin-scFv fusion proteins [270]. Furthermore, binding unfolded protein to a substrate impairs the physical interaction amongst single molecules and their refolding can be performed in a sort of "independent micro-environment" in which aggregation-driving contacts are prevented. Optimization is necessary with respect to salt concentrations to avoid protein-matrix interactions. On-column refolding was successfully performed by using metal affinity chromatography, as in the case of ribonuclease A [260], chemokines [271], and α-glucosidase [272], ion-exchange substrates, as for bovine serum albumin [273] and alfa-lactalbumin [274], or by using zeolite [275,276].

Different classes of proteins can contribute to an increase in the refolding yields of native proteins (Figure 1). Thioredoxin supported the productive refold of both reduced denatured and oxidized but incorrectly paired disulfides of pancreatic RNase, citrate synthase and alpha-glucosidase [195,277]. The peptidyl-prolyl isomerase was beneficial for the refolding of Fab fragments [278], whilst the disulfide isomerase, alone or in combination with quiescin-sulphydryl oxidase and glutaredoxin, facilitated the oxidative folding of ribonuclease A and riboflavin binding protein [279-281]. The fusion to the chaperone SlyD had a positive effect on the refolding of the ectodomain E1 of *Rubella *virus [282]. DsbA, peptidyl-prolyl-isomerase, and GroEL minichaperone were immobilized on an agarose gel to refold the scorpion toxin Cn5 [283], immobilized DsbA, DsbC and GroEL minichaperone were effective in refolding single-chain fragments [284], disulfide isomerase was beneficial for ribonuclease and lysozyme refolding [285], and DnaK in combination with trigger factor or disulfide isomerase helped the functional folding of scFv alone and fused to a toxin domain [286,287]. The availability of ClpB and DnaK/DnaJ/GrpE improved the refolding efficiency of the cysteine-rich protein gloshedobin [288]. Finally, high-pressure has also been used to prevent the formation of nonnative disulfide bonds [289].

## Conclusion

Often, when very heterogeneous molecules as the proteins are handled, it seems that the only possible approach towards optimization of the production process is the trial-and-error strategy. However, the comprehension of the physiological mechanisms involved in the protein folding and aggregation allowed for more rational solutions that simplified the selection of the conditions to be used in the trial panel (Figure 2).

Robust tools, such as oxidizing mutant strains or plasmids for the overexpression of chaperones and foldases, are now available and several successful expression alternatives have been described. There is still no certainty that a specific disulfide-bond dependent protein will be expressed in a functional form in bacteria, but a series of rational approaches can easily be compared. Once optimized the construct DNA sequence and the expression conditions, the main choice is between cytoplasmic and periplasmic folding and accumulation of the target protein. It will guide the selection of opportune leader sequences and suitable bacteria strains. Finally, the purification strategy will request further modification if the proteins are to be recovered from inclusion bodies, total lysate, periplasmic fractions, or culture medium.

At the end of this review, it becomes apparent that there is a large amount of information missing concerning the accurate description of the molecular mechanisms involved in protein folding, translocation and molecular quality control. There is also a huge gap between the theoretical knowledge and the results of protein production, as these are only sometimes in agreement with the hypothetical expectations and the apparent irrationality of some successful conditions may seem as if it were a "magical" activity. In contrast, what we are missing is a coherent system for the comparison of experimental data that remain anecdotal unless precisely annotated. Our understanding of why some conditions work in one case, but not in another, will substantially increase when a sufficient amount of homogeneous and comparable data will be available for bioinformatic analyses [290]. Data organization and their direct accessibility would help in extrapolating the information necessary to improve the identification of optimal strategies for each specific class of polypeptides. Therefore, the future collective endeavor should be addressed at rationalizing rather than accumulating data. One example to follow for future attempts might be the public repository Refold database  that lists the successful methods for refolding of proteins [291].

## Competing interests

The author declares that they have no competing interests.
